# Knockdown of DLK4 inhibits non-small cell lung cancer tumor growth by downregulating CKS2

**DOI:** 10.1515/biol-2022-0720

**Published:** 2023-09-19

**Authors:** Zongren Wan, Jipeng Wang, Qing Liu, Dan Yang, Pengling Li, Lixin Wang

**Affiliations:** Department of Respiratory and Critical Care Medicine, The Affiliated Huaian No. 1 People’s Hospital of Nanjing Medical University, Huanghe West Road, Huaiyin District, Huai‘an City, Jiangsu Province, 223300, China

**Keywords:** non-small cell lung cancer, DLX4, YB-1, CKS2, cell cycle

## Abstract

Non-small cell lung cancer (NSCLC) accounts for 85% of all lung cancer cases and is considered as the most common type of cancer. DLX4 was originally identified as a β-globin gene suppressor in red blood cells, which plays critical roles in several types of cancers. However, the role and related mechanism of DLX4 in NSCLC are still unclear. The study aimed to uncover the expression of DLX4 in human NSCLC cells and tissues, reveal its possible role in NSCLC, and investigate the underlying mechanisms. Immunoblot and TCGA database were used to detect the expression of DLX4 in human NSCLC cells and tissues. CCK-8, colony formation, and FCM assays were conducted to detect the effects of DLX4 on the viability and cell cycle of NCI-H2170 and A549 cells. Immunoblot assays were further performed to investigate the possible mechanism underlying DLX4 affecting the growth of NSCLC. We revealed that knockdown of DLX4 inhibited NSCLC cell proliferation. We further revealed that DLX4 knockdown induced the NSCLC cell cycle arrest. Our results further showed that downregulation of DLX4 suppressed YB-1 expression, which further suppressed CKS2 expression, thereby suppressing tumor growth of NSCLC. In conclusion, DLX4 has the potential to serve as a promising drug for NSCLC treatment.

## 1 Introduction

Lung cancer is one of the leading causes of cancer-related death in humans and is considered to be one of the most common types of cancers [1]. Non-small cell lung cancer (NSCLC) accounts for 85% of all lung cancer cases, of which adenocarcinoma (ADE) and squamous cell carcinoma (SCC) are the main histopathological types [2,3]. In the past several years, despite the emergence of promising therapies, surgery is not a viable option and there is no radical treatment for most patients with distant metastases [4]. Given the heterogeneity and high metastasis of lung cancer, targeted therapy may be a potentially effective treatment [5]. It is of great significance to explore the potential mechanism of NSCLC tumorigenesis and development and to search for biomarkers for lung cancer-targeted therapy with specific therapy drugs [5,6].

DLX4, a member of the DLX family, was originally identified as a β-globin gene suppressor in red blood cells [7]. A previous study showed that DLX4 is not necessary for skin development and homeostasis [8]. DLX4 is expressed in various cancers, and it has been reported that the expression level of BP1, a homeodomain-containing isoform of DLX4, is significantly higher in NSCLC than that in normal tissue samples [9]. Y-box binding protein 1 (YB-1) binds to an inverted CCAAT box [10]. YB-1 also plays a critical role in the progression of lung cancer [10]. Studies have confirmed that DLX4 promotes proliferation, cell cycle arrest, and invasion of carcinoma cells, and the expression of YB-1 is decreased in DLX4 depletion cells, while the expression of YB-1 is increased in DLX4 overexpression cells [11]. YB-1 overexpression reversed the effects of DLX4 knockdown on NPC cell proliferation, cell cycle arrest, and invasion [11]. YB-1 knockdown has been reported to reduce cyclin-dependent kinase subunit 2 (CKS2) expression [11]. Knockdown of CKS2 can inhibit cell proliferation, induce cell cycle arrest, and increase the expression of P53, P21, and phosphatase and tensin homolog deleted on chromosome 10 (PTEN) [11]. However, the role and related mechanism of DLX4 in NSCLC and the possible mechanism are still unclear.

This study aimed to detect the expression of DLX4 in human NSCLC cells and tissues and reveal its possible role in NSCLC, and the underlying mechanisms. In this study, we used NSCLC cell lines NCI-H2170 and A549 cells to reveal the role of DLX4 in lung cancer. We found that downregulation of DLX4 inhibited YB-1 expression, which in turn suppressed CKS2 expression, thus inhibiting tumor growth.

## 2 Materials and methods

### 2.1 Cell culture and transfection

Human lung cell line BEAS-2B (control cells), and four NSCLC cell lines, including NCI-H2170, A549, and NCI-H1975 cells, were bought from ATCC and cultured in Dulbecco's Modified Eagle Medium containing 10% fetal bovine saline (Gibco, Rockville, MD, USA) with 1% penicillin/streptomycin in an incubator at 37°C and 5% CO_2_. The two DLX4 shRNAs (#1 and #2) and the pcDNA3.1-CKS-2 and pcDNA3.1-YB-1 plasmids were constructed in our lab and transfected into cells via lipofectamine 2000. pcDNA3.1-vector or control shRNAs were provided as the corresponding control groups. The experiments were performed in the lab at the Department of Respiratory and Critical Care Medicine in our hospital.

### 2.2 CCK-8 assays

NCI-H2170 and A549 cells were seeded into 96-well plates. With different transfections, NCI-H2170 and A549 cells were treated with CCK-8 solution for 3 h, and then OD value of the different groups was evaluated at a wavelength of 450 nm.

### 2.3 Colony formation assay

NCI-H2170 and A549 cells with different transfections were seeded into six-well plates for 14 days. Then, cells were washed with phosphate-buffered saline (PBS), fixed with 4% paraformaldehyde, photographed under a microscope, and the number of colonies was counted (Olympus, Ishikawa, Japan).

### 2.4 Flow cytometry

NCI-H2170 and A549 cells were seeded into six-well plates for different treatments. NCI-H2170 and A549 cells were first collected into 1.5 mL EP tubes and then incubated with propidium iodide for 15 min and finally assessed by flow cytometry analysis.

### 2.5 Western blot

Cell lysates were extracted with radio immunoprecipitation assay buffer (Beyotime, China), added with protease and phosphatase inhibitors, and the protein concentration was measured by a BCA Protein Assay kit (Beyotime, China). To separate proteins with different molecular weights, 7.5 and 10% sodium dodecyl sulfate–polyacrylamide gel electrophoresis were used. Then, the proteins were transferred to polyvinylidene difluoride membranes. After blocking with 5% skim milk in PBS, the membranes were incubated with primary antibodies against DLX4 (1:500; Abcam, Cambridge, UK), p53 (1:500; Abcam, Cambridge, UK), p21 (1:500; Abcam, Cambridge, UK), PTEN (1:500; Abcam, Cambridge, UK), CKS-2 (1:500; Abcam, Cambridge, UK), YB-1 (1:500; Abcam, Cambridge, UK), and GAPDH (1:2,000; Santa Cruz, California, USA) for 16 h at 4°C. Finally, the membranes were incubated with horseradish peroxidase-conjugated secondary antibodies (1:500; Beyotime, China) and evaluated by Molecular Imager ChemiDoc XRS + System (Bio-Rad, Philadelphia, PA, USA).

### 2.6 Statistical analysis

SPSS software was used for statistical analysis. Unpaired two-tailed Student’s *t* test was used to perform statistical analyses. Data were presented as mean ± standard error of the mean. Differences were considered statistically significant when *p* < 0.05.

## 3 Results

### 3.1 DLX4 was highly expressed in human NSCLC tissues and cells

**Figure 1:**
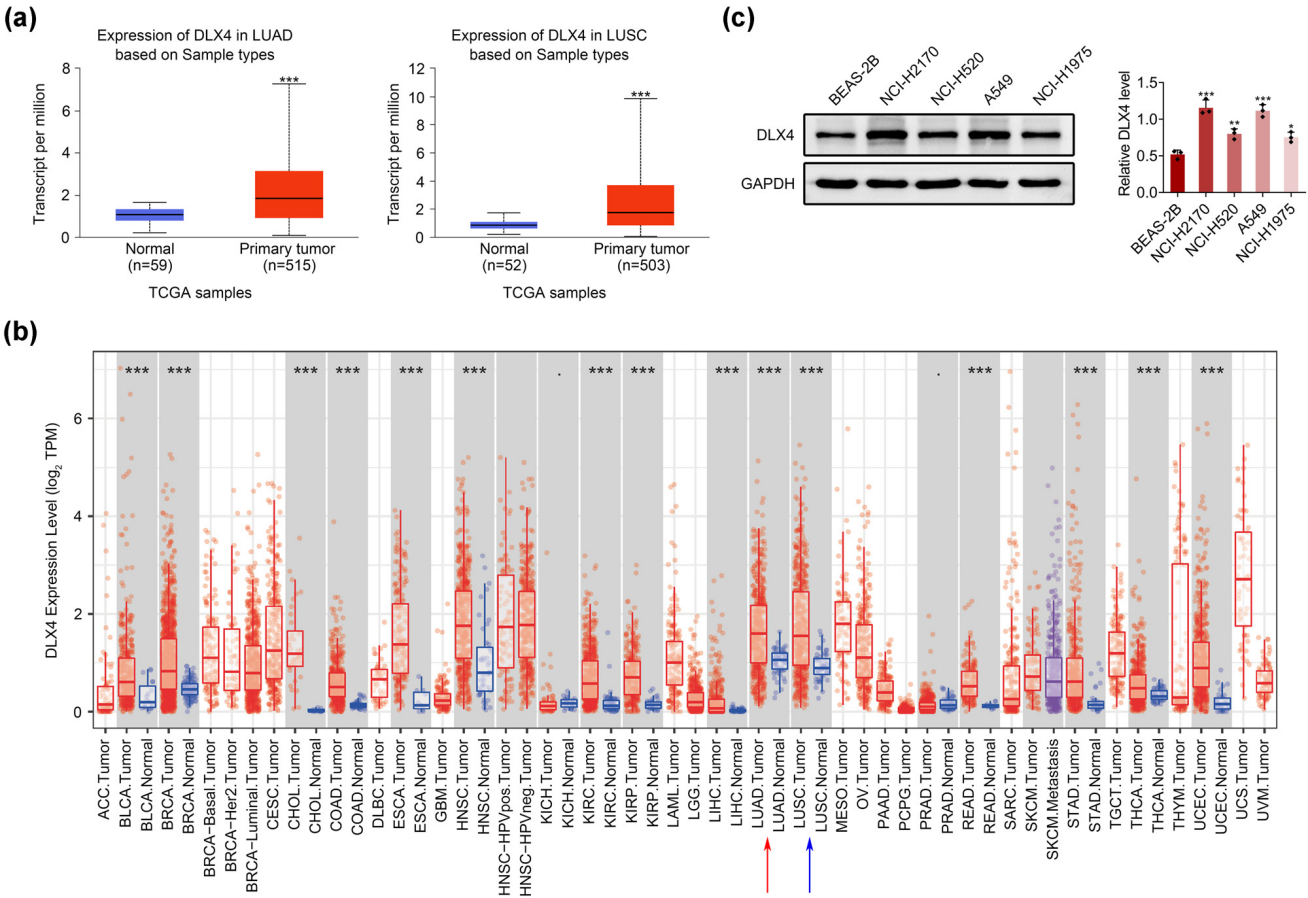
DLX4 was highly expressed in human NSCLC tissues and cells. (a) TCGA database showed the transcript per million of DLX4 in 515 LUAD and 503 LUSC tissues compared to the corresponding normal tissues. (b) The TIMER database showed the expression levels of DLX4 in different types of cancer tissues and corresponding normal tissues. (c) Immunoblot showed the expression of DLX4 in human lung cell line BEAS-2B, and four types of NSCLC cell lines, including NCI-H2170, A549, and NCI-H1975. Data were presented as mean ± SD, *p < 0.05, **p < 0.01, ***p < 0.001.

To uncover the role of DLX4 in NSCLC progression, we first detected its expression in NSCLC tissues via bioinformation analysis. Through TCGA database, we revealed that DLX4 was highly expressed in lung cancer tissues compared to normal tissues (Figure 1a), with a high transcript per million (Figure 1a). We further investigated the expression of DLX4 in different types of cancers through the database. We revealed that DLX4 was highly expressed in multiple types of cancers, such as breast cancer and colon cancer (COAD) (Figure 1b). Notably, it was also confirmed that DLX4 was highly expressed in NSCLC tissues (Figure 1b). Subsequently, we detected the expression of DLX4 in human lung cell line BEAS-2B, and four types of NSCLC cell lines, including NCI-H2170, A549, and NCI-H1975 cells. Through Immunoblot, we noticed that DLX4 was highly expressed in human NSCLC cell lines (Figure 1c). These data confirmed that DLX4 was highly expressed in human NSCLC tissues and cells.

### 3.2 DLX4 knockdown suppressed the viability of NSCLC cells

**Figure 2:**
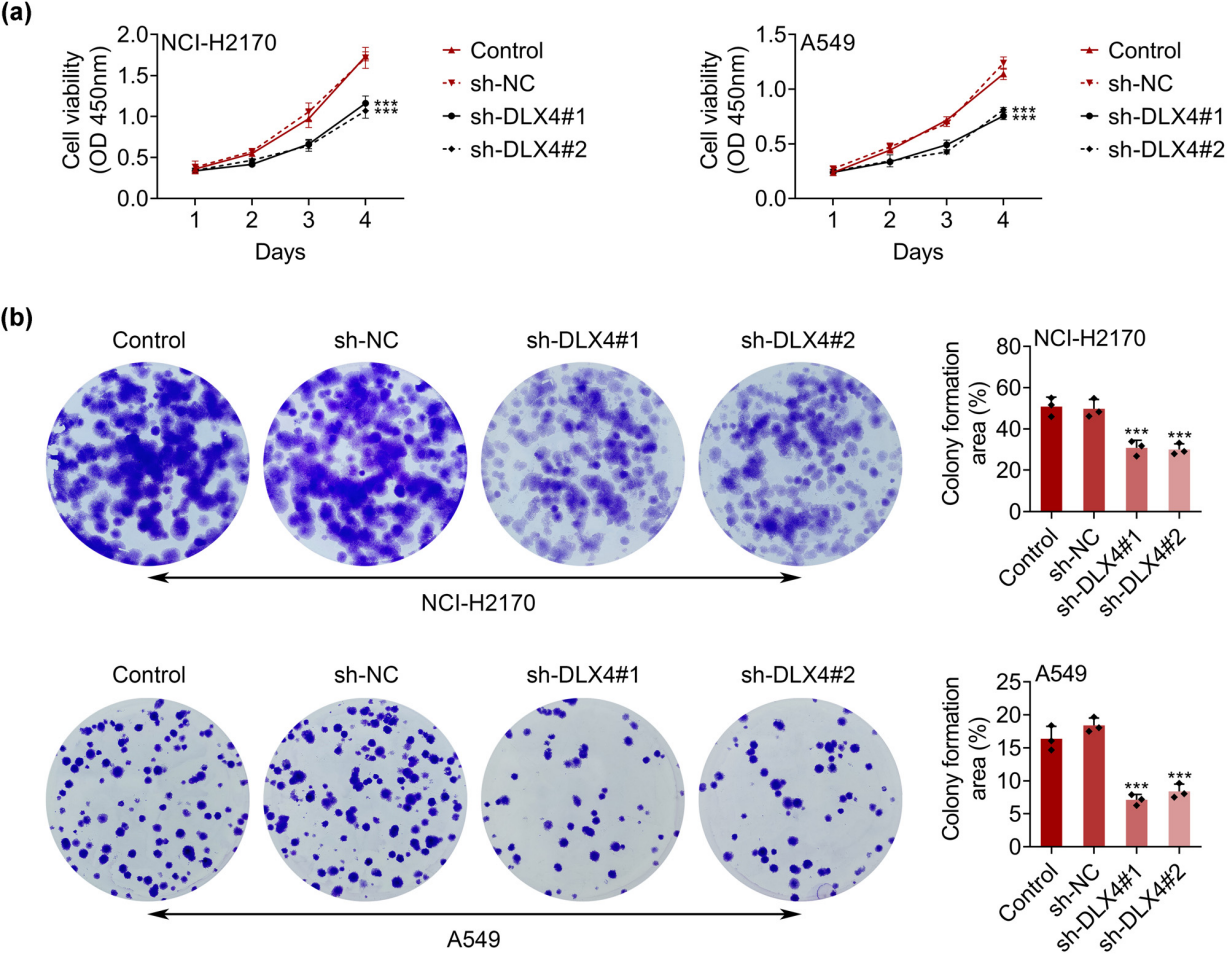
DLX4 depletion suppressed the viability of NSCLC cells. (a) CCK-8 assays showed the OD value at 450 nm wavelength in NCI-H2170 and A549 cells upon the indicated transfection. (b) Colony formation assays showed the colony numbers of NCI-H2170 and A549 cells upon the indicated transfection. Data were presented as mean ± SD, ***p < 0.001. NC: negative control.

Since DLX4 was highly expressed in NSCLC, we then detected its effects on the growth of NSCLC cells. We revealed that DLX4 knockdown by transfection of two types of DLX4 shRNAs suppressed the viability of NSCLC cells, which was confirmed with the decreased OD450 value through CCK-8 assays (Figure 2a). Furthermore, through colony formation assays, we revealed that DLX4 knockdown suppressed the proliferation of both NCI-H2170 and A549 cells, with decreased colony numbers (Figure 2b). Therefore, these results suggested that DLX4 knockdown suppressed the viability and growth of NSCLC cells.

### 3.3 DLX4 knockdown induced cell cycle arrest of NSCLC cells

**Figure 3:**
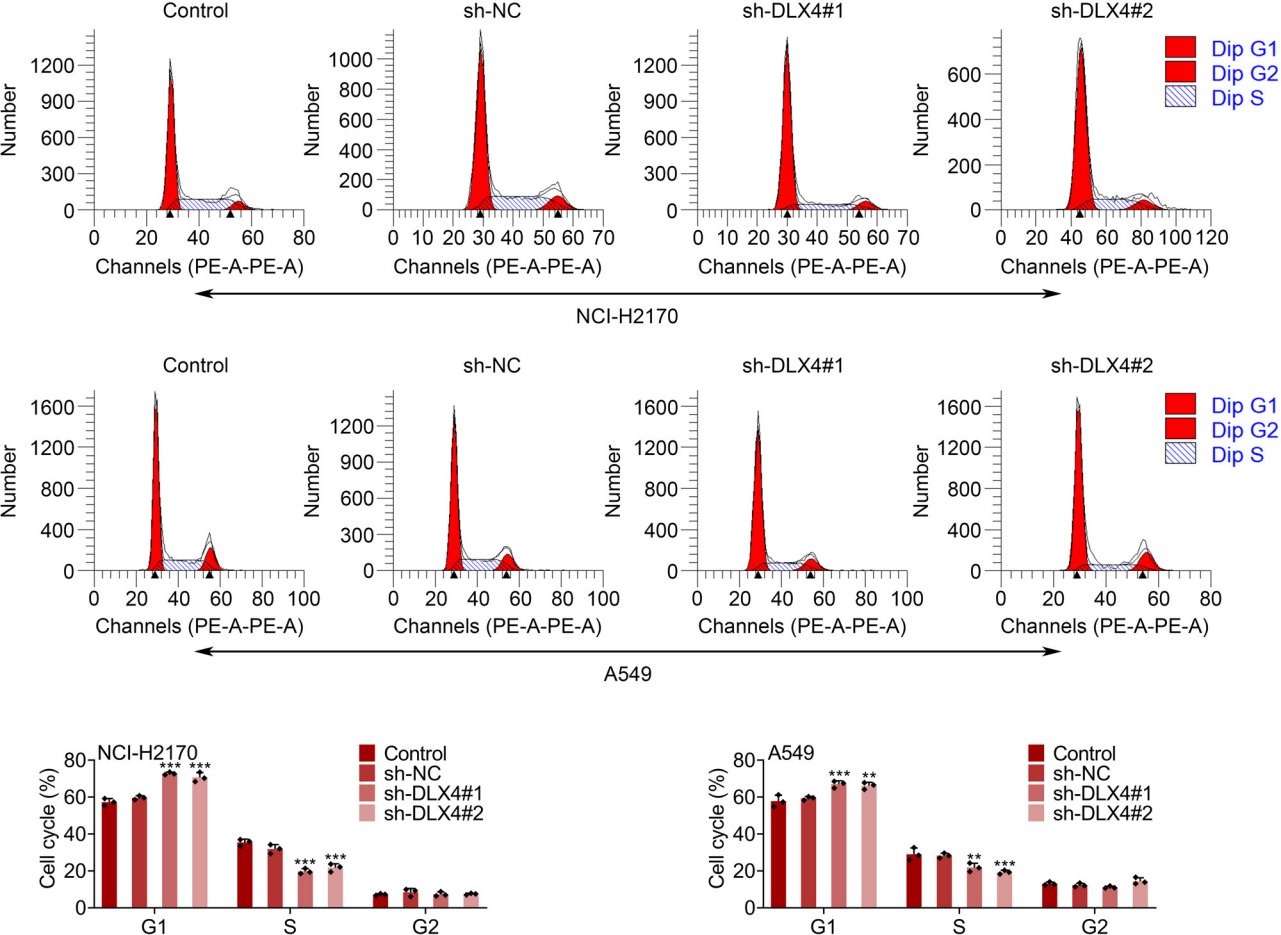
DLX4 knockdown induced cell cycle arrest of NSCLC cells. FCM assays showed the percentage of cells at G1, S, and G2/M phase of NCI-H2170 and A549 cells upon the indicated transfection. Data were presented as mean ± SD, **p < 0.01, ***p < 0.001. NC: negative control.

Since DLX4 knockdown suppressed the viability of NSCLC cells, we further investigated whether downregulation of DLX4 affected the cell cycle of NSCLC cells. Through FCM assays, we found that DLX4 knockdown induced cell cycle arrest of NCI-H2170 and A549 cells, with an increased percentage of cells in G1 phase and decreased cells in S phase, suggesting G1/S phase arrest (Figure 3). Therefore, DLX4 knockdown induced cell cycle arrest of NSCLC cells.

### 3.4 Effect of DLX4 knockdown on p53, p21, and PTEN

**Figure 4:**
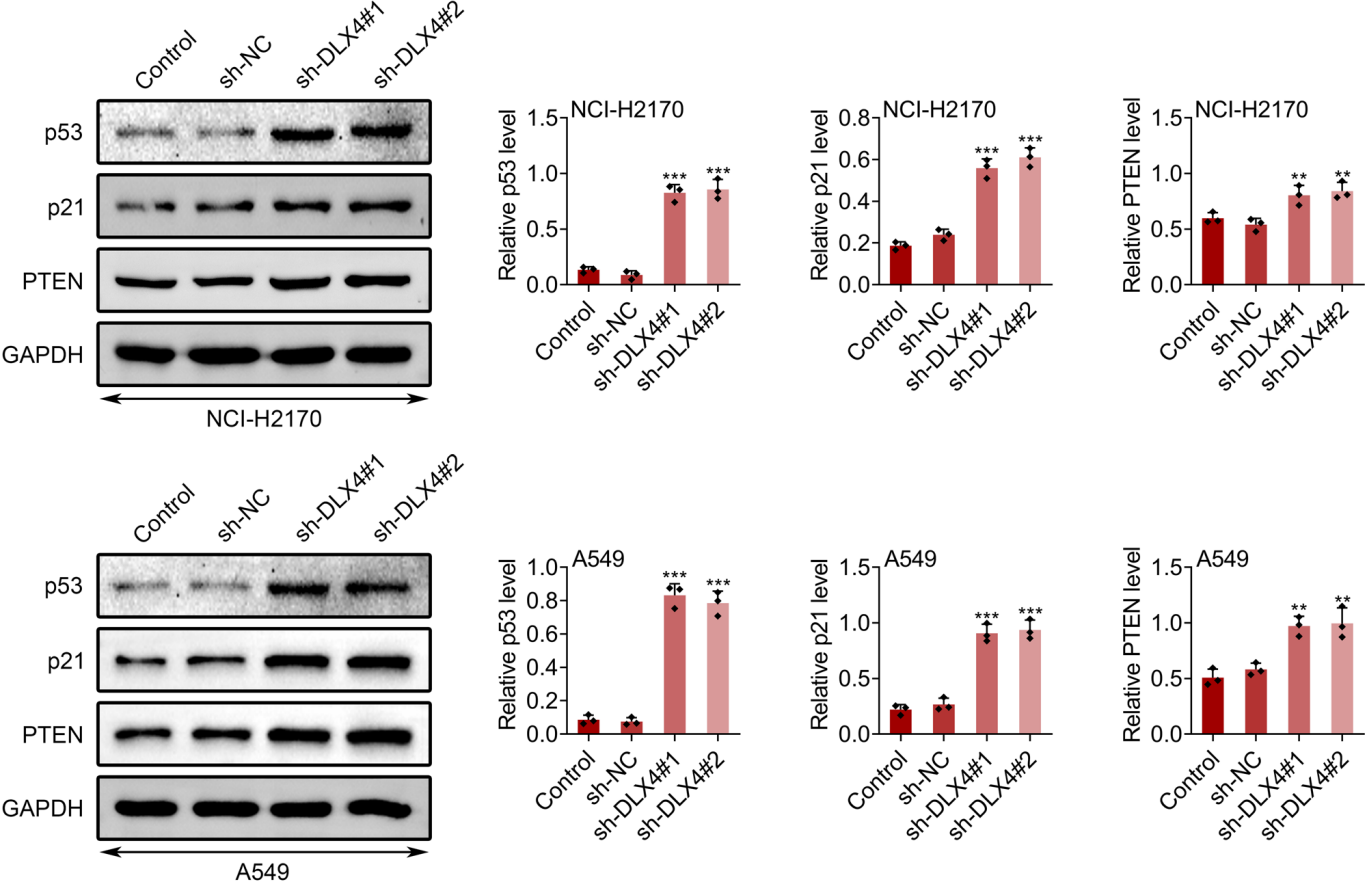
Effect of DLX4 knockdown on p53, p21, and PTEN. Immunoblot showed the expression of p53, p21, and PTEN in NCI-H2170 and A549 cells upon the indicated transfection. Data were presented as mean ± SD, *p < 0.05, **p < 0.01, ***p < 0.001. NC: negative control.

Furthermore, we detected the effects of DLX4 knockdown on p53 pathway in NSCLC cells through Immunoblot. We detected the expression of several downstream proteins of CKI (casein kinase 1), including p53, p21, and PTEN. We revealed that the knockdown of DLX4 increased the expression of p53, p21, and PTEN in NCI-H2170 and A549 cells, suggesting that DLX4 knockdown increased CKI in NSCLC cells (Figure 4).

### 3.5 DLX4 depletion suppressed the expression of YB-1 and CKS-2 in NSCLC cells

**Figure 5:**
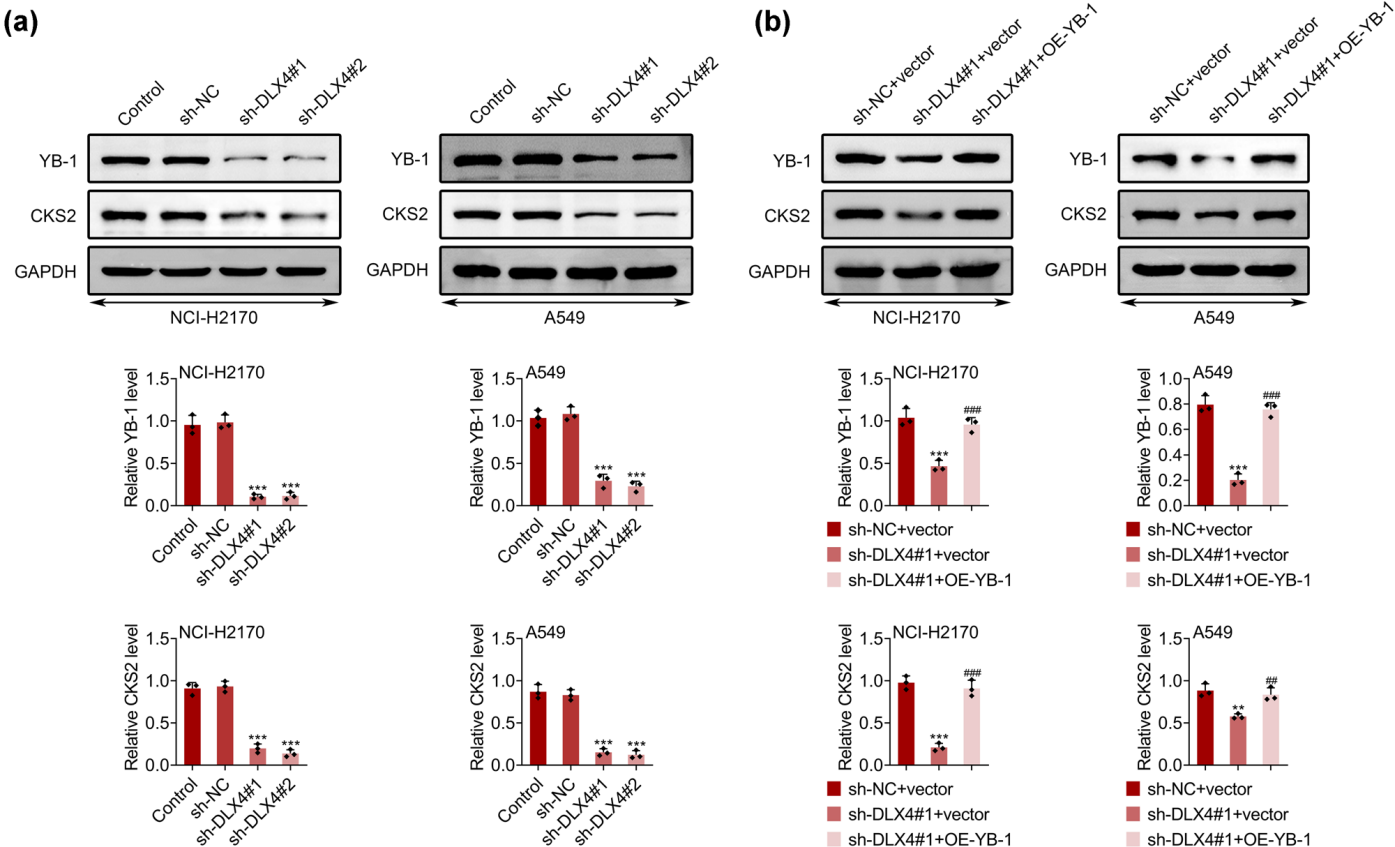
DLX4 depletion suppressed the expression of YB-1 and CKS-2 in NSCLC cells. (a) Immunoblot showed the expression of YB-1 and CKS-2 in NCI-H2170 and A549 cells upon the indicated transfection. (b) Immunoblot showed the expression of YB-1 and CKS-2 in NCI-H2170 and A549 cells upon the indicated transfection. Data were presented as mean ± SD, **p < 0.01, ***p < 0.001 vs sh-NC-vector, ^##^ *p* < 0.01, ^###^ *p* < 0.001 vs shDLX4#1 + vector. NC: negative control.

In the previous study, DLX4 affected the progression of cancer by mediating the expression of YB-1 and CKS-2 [11]. Therefore, here we detected the effects of DLX4 on the expression of YB-1 and CKS-2 in NSCLC cells. Through Immunoblot, we confirmed that the knockdown of DLX4 suppressed the expression of both YB-1 and CKS-2 in NCI-H2170 and A549 cells, suggesting the suppression of YB-1/CKS-2 axis in NSCLC cells (Figure 5a). We further found that overexpression of YB-1 reversed the suppressed YB-1 and CKS-2 expression in NCI-H2170 and A549 cells (Figure 5b). Therefore, DLX4 knockdown suppressed the expression of YB-1 and CKS-2 in NSCLC cells.

### 3.6 Knockdown of DLX4 inhibited NSCLC progression by downregulating CKS2

**Figure 6:**
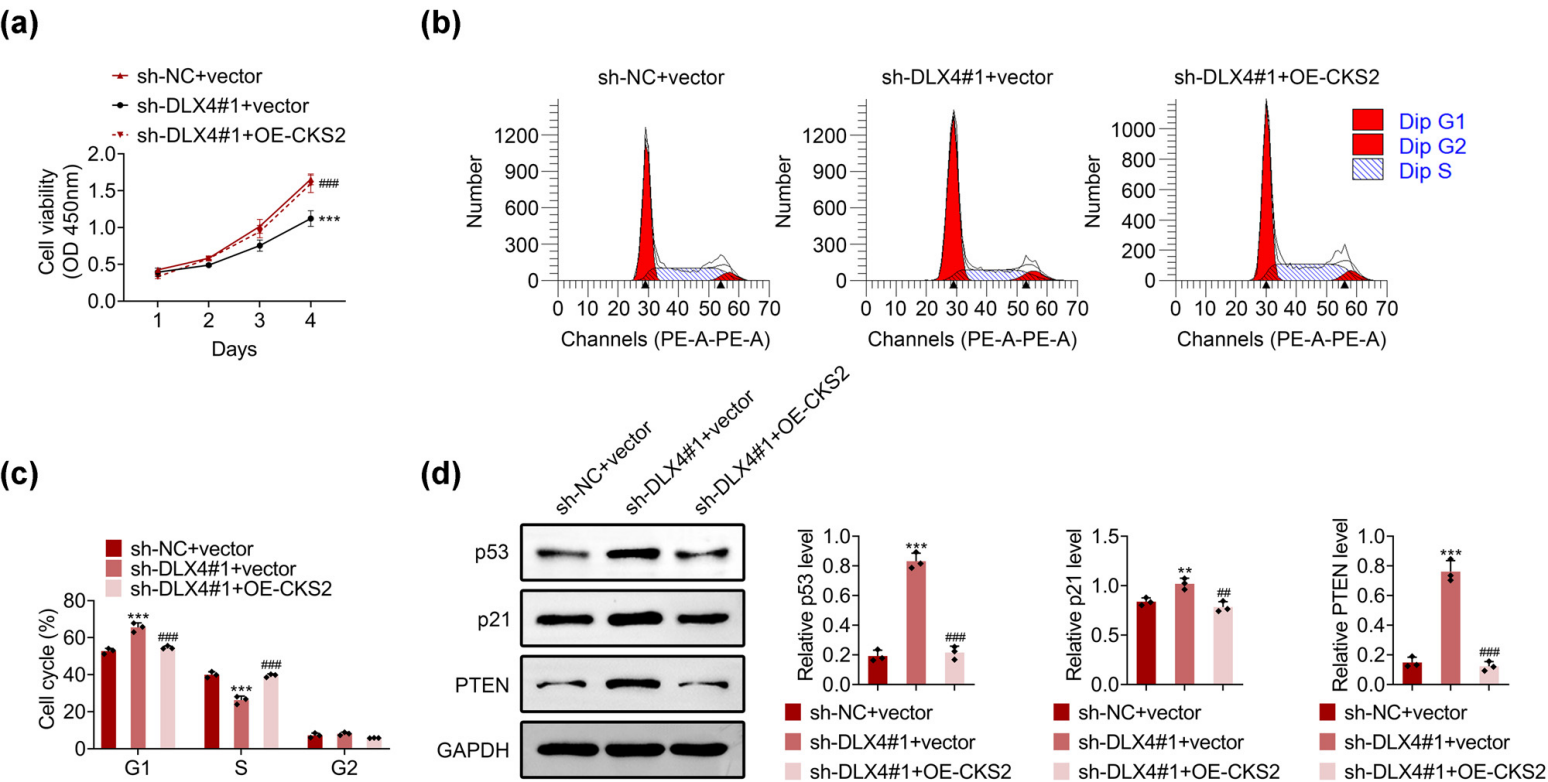
Knockdown of DLX4 inhibited NSCLC progression by downregulating CKS2. (a) CCK-8 assays showed the OD value at 450 nm wavelength in NCI-H2170 cells upon the indicated transfection. (b) Colony formation assays showed the colony numbers of NCI-H2170 cells upon the indicated transfection. (c) Immunoblot showed the expression of p53, p21, and PTEN in NCI-H2170 cells upon the indicated transfection. (d) Immunoblot showed the expression of p53, p21, and PTEN in NCI-H2170 cells upon the indicated treatment. Data were presented as mean ± SD, ***p < 0.001 vs sh-NC-vector, ^##^ *p* < 0.01, ^###^ *p* < 0.001 vs shDLX4#1 + vector. NC: negative control.

We then investigated whether DLX4 affected NSCLC progression by targeting CKS2. Through CCK-8 assays, we noticed that DLX4 knockdown suppressed the viability of NCI-H2170 cells, whereas overexpression of CKS2 rescued the suppression of cell viability (Figure 6a). Furthermore, through FCM assays, we revealed that DLX4 knockdown induced the G1/S phase arrest of NCI-H2170 cells, and overexpression of CKS2 reversed the cell cycle arrest (Figure 6b). We further performed Immunoblot, and the results showed that CKS2 overexpression reversed the downregulated expression of p53, p21, and pTEN in NCI-H2170 cells. These results demonstrated that knockdown of DLX4 inhibited NSCLC progression by downregulating CKS2.

## 4 Discussion

NSCLC, including SCC and ADE, has slower growth and division than small cell carcinoma, and its metastasis is relatively late [12]. In recent years, the treatment of NSCLC mainly includes surgical resection, radiotherapy, and chemotherapy. However, due to its high metastasis and high recurrence rate, these treatments are difficult to achieve satisfactory results [12]. Targeted therapy for NSCLC has attracted more and more attention, and screening its key targets is a good choice for improving the prognosis of patients with advanced stage [13,14,15]. In this study, we revealed the high expression of DLX4 in NSCLC cells, and our results suggest that DLX4 has the potential to serve as a promising target of NSCLC.

Using multiple *in vitro* assays, such as CCK-8 and colony formation and FCM assays, our results demonstrated that DLX4 knockdown suppressed cell viability and stimulated the cell cycle arrest of two types of NSCLC cells, including NCI--H2170 and A549 cells, suggesting that DLX4 could affect the progression of NSCLC. In fact, the role of DLX4 has been widely revealed in several types of cancers, such as renal cancer and endometrial cancer [16]. DLX4 hypermethylation was a prognostically adverse indicator in acute myeloid leukemia [17]. DLX4 could contribute to the progress of clear cell renal cell carcinoma by stimulating EMT [18]. Another study reported that DLX4 facilitated the progression of nasopharyngeal carcinoma via upregulation of YB-1, which was similar to our findings [11]. In addition, DLX4 overexpression could promote cell viability and motility and predict poor prognosis in endometrial cancer [16]. These studies, combined with our findings, confirmed that DLX4 has the potential to serve as a critical cancer target.

CKS2 is essential for regulating cell cycle progression [19]. CKS2 is involved in cell cycle control through interaction with maturation promoting factor [19]. Our previous studies have confirmed that CKS2 is highly expressed in NSCLC. Knockdown CKS2 can inhibit cell proliferation, induce cell cycle arrest, and increase the expression of P53, P21 and PTEN, suggesting that CKS2 may be biomarkers of NSCLC [20]. In this study, we revealed that DLX4 could regulate the expression of P53, P21, and PTEN via CKS2 in NSCLC cells, and we speculated that DLX4 could affect the viability and tumor growth of NSCLC by mediating CKS2.

YB-1 binds to the inverted CCAAT box [11]. YB-1 is a multifunctional protein that regulates translation [11]. It has been reported that YB-1 is highly expressed in lung cancer, and knocking down YB-1 can inhibit lung cancer cell proliferation and induce cell cycle arrest [21]. In addition, YB-1 knockdown has been reported to reduce CKS2 expression [22]. This study also revealed that DLX4 knockdown blocked the cell cycle of NPC at G1 phase, suggesting the anti-tumor effect of DLX4 knockdown on NPC [22]. The downstream target of DLX4 was YB-1, whose expression was increased by overexpression of DLX4, while decreased by knockdown of DLX4 [22]. The binding relationship between DLX4 and YB was verified by chromatin immunoprecipitation, and the result showed that DLX4 could not directly bind to the promoter of YB [22]. Interestingly, in this study, we revealed that DLX4 could affect the viability of NSCLC cells by mediating CKS2 and YB-1 expression. We believed that the CKS2/YB-1 axis plays a critical role in NSCLC progression and may serve as a promising target of NSCLC.

In summary, this study suggested that the knockdown of DLX4 suppressed the viability of NSCLC cells and stimulated NSCLC cell cycle arrest. Downregulation of DLX4 inhibited YB-1 expression, and suppressed CKS2 expression, thereby inhibiting tumor growth of NSCLC. Therefore, our findings suggested that DLX4 may serve as a therapeutic target for the treatment of NSCLC.
